# Esophageal peripheral T-cell lymphoma treated with radiotherapy: A case report

**DOI:** 10.1097/MD.0000000000024455

**Published:** 2021-01-29

**Authors:** Qiujing Zhang, Chengxiang Liu, Zining Liu, Menghan Liu, Chao Xie, Jinsong Zheng, Congcong Han, Dexian Zhang, Jianjun Zhang, Shuai Fu, Jie Liu

**Affiliations:** a Department of Oncology, Shandong Cancer Hospital and Institute, Shandong First Medical University and Shandong Academy of Medical Sciences; b Department of Oncology, Jinan Jigang Hospital; c Department of Oncology, The Third Affiliated Hospital of Shandong First Medical University, Jinan, Shandong, China; d Basic Medicine College, Shandong First Medical University, Taian; e Department of imaging; f Department of Pathology, Shandong Cancer Hospital and Institute, Shandong First Medical University and Shandong Academy of Medical Sciences, Jinan, Shandong, China.

**Keywords:** case report, cyclophosphamide–Adriamycin–vincristine–prednisone, peripheral T-cell lymphoma, not otherwise specified, radiotherapy

## Abstract

**Rationale:**

The clinical prognosis of peripheral T-cell lymphoma, not otherwise specified (PTCL-NOS) patients is poor. Therefore, effective treatment is still a challenge at present. Moreover, little is known about the value of radiotherapy in the treatment of PTCL-NOS.

**Patient concerns:**

A 55-year-old male patient with eating difficulties and progressive exacerbation for 3 months was diagnosed as non-Hodgkin's lymphoma. Airway compression occurred after 2 cycles of first line treatment with cyclophosphamide–Adriamycin–vincristine–prednisone regimen, radiotherapy (48Gy/24f) was given as the second line therapy.

**Diagnosis:**

After radiotherapy, the patient complained that mild intermittent dysphagia still existed. Endoscopic biopsy of the upper digestive tract confirmed necrotic material and superficial squamous epithelial mucosa, suggesting esophageal stricture after radiotherapy, which was indistinguishable from tumor residue.

**Interventions:**

The patient received anti-inflammatory treatment outside the hospital and did not receive any other special treatment.

**Outcomes:**

The symptoms of dysphagia disappeared and the focus showed complete response (CR). As of October 1, 2020, the patient has been diagnosed with PTCL-NOS for more than 57 months and the overall survival (OS) have not been achieved.

**Lessons:**

Radiotherapy has obvious and rapid anti-tumor effect on cyclophosphamide–Adriamycin–vincristine–prednisone refractory PTCL-NOS. At the same time, hollow organs after radiotherapy can lead to lumen stenosis and the symptoms of suspected recurrence which is difficult to distinguish only from the imaging findings.

## 1 Introduction

Peripheral T-cell lymphoma (PTCL) is a kind of heterogeneous lymphoma with low incidence (accounting for 10% of non-Hodgkin's lymphoma) but highly invasive.^[1]^ The clinical prognosis of patients with PTCL is poor, especially in patients with relapse or refractory, the median OS is less than half a year.^[2]^ The T-cell project launched by International T-Cell Lymphoma Project also confirmed this finding. In this study, the median survival after relapse (SAR) of refractory patients was only 5 months, and 3-year SAR rate was only 21%.^[3]^ Although new drugs have been approved for the treatment of PTCL in recent years, the prognosis of patients has not been significantly improved. Anthracycline regimens – such as cyclophosphamide–Adriamycin–vincristine–prednisone, CHOP – are still recommended for first-line treatment. Unfortunately, most types of PTCL, including PTCL-NOS, do not benefit from this scheme.^[4,5]^

PTCL-NOS is an exclusive diagnosis, independent of other independent typing of PTCL can be diagnosed as PTCL-NOS, so PTCL-NOS is the most common subtype of PTCL, accounting for about 30% of all PTCL patients.^[6]^ CHOP with or without radiotherapy is most commonly used for patients with PTCL-NOS as first-line treatment, but the heterogeneity of the disease leads to poor survival (the 5-year survival rate is about 30%).^[4,7]^ To the best of our knowledge, the value of radiotherapy in the treatment of PTCL-NOS has not been clearly explained and esophageal involvement in PTCL is extremely rare.^[8]^ Here, we will report a case of esophageal PTCL who has achieved significant survival benefits from radiotherapy.

## 2 Case presentation

The patient is a 55-year-old male without any history of smoking or drinking or any special family history. On December 11, 2015, the patient came to our hospital for “eating difficulty without obvious inducement and progressive aggravation for 3 months”. Prior to this, the patient underwent a computed tomography (CT) scan and the esophageal tissue 17 to 31 cm below the upper incisor was removed for biopsy in a local hospital. Subsequently, submucosal lymphoid follicles of the same size were observed. Combined with the results of immunohistochemistry, the diagnosis of non-Hodgkin's lymphoma was not excluded. Therefore, the patient came to our hospital for treatment.

**Figure 1:**
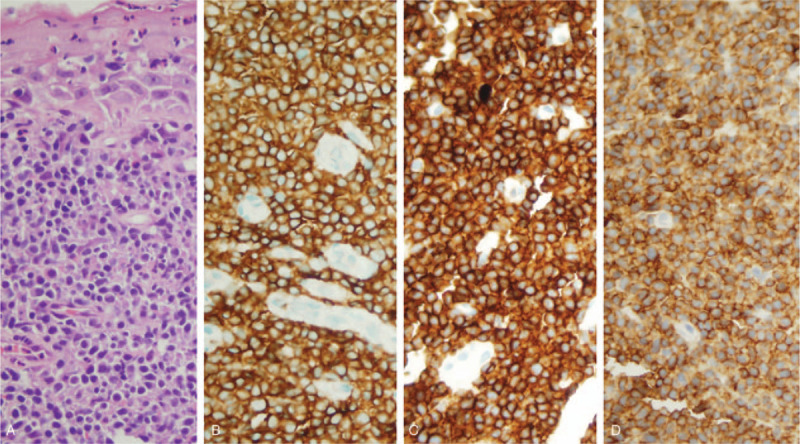
Pathological images of the patient. Panel A is the result of hematoxylin-eosin staining of the patient. Panels B, C and D display CD3+, CD7+ and CD8+, respectively (×400).

**Figure 2:**
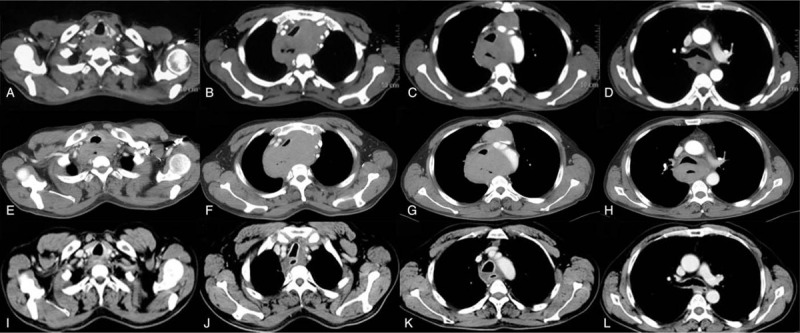
CT images of patients before and after treatment. A–D: the baseline CT of the patient; E-H: disease progression after 2 cycles CHOP regimen; I–L: the esophageal lesions disappeared after radiotherapy. CHOP = cyclophosphamide–Adriamycin–vincristine–prednisone.

After consultation with the external hospital pathology, we believed that the patient met the diagnosis of T-cell non-Hodgkin's lymphoma, and the original tissue sample immunohistochemistry findings were: CD3 (+), CD7 (+), CD8 (+), CD5 (−), CD20 (−), bcl-6 (−), mum-1 (−), CD10 (−), bcl-2 (−), Kappa (−), Lambda (−), CyclinD (−), CD56 (−), CD138 (−) (Fig. 1. tif). In addition, CT scan of the local hospital showed that the upper and middle thoracic esophageal walls and mediastinal lymph nodes were thickened and moderately uniformly enhanced (Fig. 2A-D. tif). Combined with the pathological types, clinical manifestations and imaging data, the patient was finally diagnosed as stage IV esophageal PTCL-NOS with expression of CD3, CD7 and CD8. No obvious abnormal bone marrow image was found in bone marrow puncture.

On admission, the patient had already developed dysphagia without heartburn, epigastric pain, cough, expectoration, dyspnea, chest pain, fever and night sweats. The Eastern American Cancer Cooperation Group (ECOG) performance status of the patient scored 2 points. Laboratory hematology and blood biochemical tests revealed that leucocyte 13.37/L (3.5–9.5/L), neutrophils 11.46/L (1.8–6.8/L), neutrophils percent 85.7% (40%–75%), and albumin / globulin 1.01 (1.2–2.4). No abnormality was found in lymphocyte count, platelet count, serum biochemical test, anti-HIV antibody and hepatitis virus detection, Furthermore, there was no palpable obvious lymphadenopathy, ascites or organomegaly. According to Ann Arbor staging, the disease was grade IIE, the age-adjusted international prognostic index (aa-IPI) was high-intermediate and the prognostic index for PTCL- NOS (PIT) belongs to Group 2.

**Figure 3:**
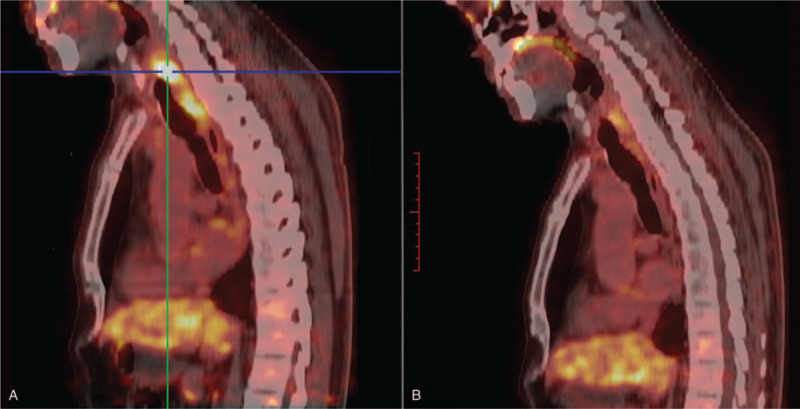
PET image of esophageal inflammation after radiotherapy. Panel A showed the hypermetabolic foci after radiotherapy in esophagus, panel B is the PET manifestation after symptomatic treatment. PET = positron emission tomography.

After 2 cycles of first-line chemotherapy with CHOP regimen (Epirubicin 80 mg d1 + cyclophosphamide 0.6 g d1,8 + vincristine 2 mg d8 + prednisone 100 mg po d1–5), dysphagia worsened and dyspnea occurred. Enhanced CT showed enlarged lesions protruding from the esophagus to the anterior mediastinum, resulting in uniform tracheal compression (Fig. 2E–H. tif). In order to reduce the life-threatening compression of local tumors, the patients received radiotherapy with a total dose of 48Gy/24f. Upon admission to the hospital after radiotherapy, the patient reported a significant remission of dyspnea, which was later confirmed by enhanced CT scans: the tumor disappeared (the efficacy was evaluated as CR according to RECIST criteria) (Fig. 2I–L. tif). Subsequently, the patient received gemcitabine 1.6 g d1,8 + oxaliplatin 200 mg d2 for 6 cycles, during which CT showed no signs of recurrence. However, according to the patient's report, mild intermittent dysphagia still existed. Positron emission tomography (PET) revealed hypermetabolic lesions in the esophagus at the thoracic inlet, which were considered a residual tumor (Fig. 3A. tif). Therefore, we removed part of the tissue through upper gastrointestinal endoscopy for biopsy. Subsequently, the hypermetabolic lesions were pathologically confirmed as necrotic material and superficial squamous epithelial mucosa. This suggests that esophageal stenosis due to tissue edema caused by inflammation after radiotherapy is difficult to distinguish from symptoms of tumor recurrence. After 4 months of anti-inflammatory treatment, the symptoms of dysphagia disappeared and PET showed that the hypermetabolic foci disappeared completely (Fig. 3B. tif). This further confirms that the hypermetabolic lesions shown by imaging after radiotherapy are not necessarily caused by tumor remnants.

As of October 01, 2020, the patient has been diagnosed with PTCL-NOS for more than 57 months, the progression-free survival (PFS) has reached 27 months and the OS has not reached. Now, the patient is undergoing continuous follow-up. According to the regularity of follow-up, it is concluded that there was better intervention adherence of this patient. In addition, the patient did not have any treatment-related adverse events (TRAE) during radiotherapy.

## 3 Discussion

It is well known that painless enlargement of superficial lymph nodes is the most common clinical manifestation of PTCL-NOS and any other organ may also be involved (including bone marrow (22%), liver, spleen and skin, with nodal disease),^[7]^ but esophageal involvement is very rare.^[8]^ We described a patient with refractory esophageal PTCL after CHOP treatment gained long-term survival benefits from radiotherapy. Studies have found that PTCL-NOS patients are still mainly treated with anthracycline regimens such as CHOP and rarely receive radiotherapy,^[9]^ but these treatments rarely enable PTCL-NOS patients to achieve long-term disease-free survival. Therefore, more effective treatment is still a challenge at present.

In recent years, it has been reported that the application of radiotherapy may improve the survival rate of PTCL-NOS patients. In this study, 35 patients with early PTCL-NOS were randomly divided into two groups: chemotherapy alone and chemotherapy combined with radiotherapy. The results showed that the 3-year OS rate of the combined treatment group was 49.7%. The intervention of radiotherapy significantly improved the survival of early PTCL-NOS patients.^[10]^ However, it is not clear whether patients with advanced PTCL-NOS can benefit from radiotherapy.

In this case of advanced PTCL-NOS, the large mass of CHOP refractory esophageal T-cell lymphoma was quickly eliminated after radiotherapy. So far, the patient has been diagnosed with PTCL-NOS for more than 57 months, the PFS has reached 27 months and OS has not been reached. In addition, the patient tolerated the radiotherapy and there was no TRAE that occurred during radiotherapy. This patient progressed after two cycles of CHOP treatment, and the tumor shrank rapidly after timely intervention with radiotherapy (the efficacy was evaluated as CR). However, he still felt dysphagia occasionally after radiotherapy. PET revealed hypermetabolic lesions, which we suspected to be residual tumor, but this was not the case.

Therefore, we hope that our colleagues will note that radiotherapy has a significant and rapid anti-tumor effect on CHOP refractory PTCL-NOS. Meanwhile, the radiotherapy for hollow viscera may result in post-radiotherapy stenosis manifested as obstruction symptom and high metabolic focal inflammation in PET-CT which is difficult to differentiate from residual tumors.

## Author contributions

**Conceptualization:** Qiujing Zhang, Jie Liu.

**Data curation:** Chengxiang Liu.

**Investigation:** Zining Liu, Menghan Liu, Congcong Han.

**Supervision:** Jie Liu.

**Visualization:** Chao Xie, Jinsong Zheng, Dexian Zhang.

**Writing – original draft:** Qiujing Zhang, Chengxiang Liu.

**Writing – review & editing:** Jianjun Zhang, Shuai Fu.

## Abbreviations

Abbreviations: aa-IPI = age-adjusted international prognostic index, CHOP = cyclophosphamide–Adriamycin–vincristine–prednisone, CR = complete response, CT = computed tomography, ECOG = Eastern American Cancer Cooperation Group, OS = overall survival, PET = positron emission tomography, PFS = progression-free survival, PIT = the prognostic index for PTCL- NOS, PTCL = Peripheral T-cell lymphoma, PTCL-NOS = peripheral T-cell lymphoma, not otherwise specified, SAR = survival after relapse, TRAE = treatment-related adverse event.

## References

[1] Siaghani PJ Song JY. Updates of Peripheral T Cell lymphomas based on the 2017 WHO classification. Curr Hematol Malig Rep 2018;13:25–36. 29442288 10.1007/s11899-018-0429-y

[2] Mak V Hamm J Chhanabhai M. Survival of patients with peripheral T-cell lymphoma after first relapse or progression: spectrum of disease and rare long-term survivors. J Clin Oncol 2013;31:1970–6. 23610113 10.1200/JCO.2012.44.7524

[3] Bellei M Foss FM Shustov AR. The outcome of peripheral T-cell lymphoma patients failing first-line therapy: a report from the prospective, International T-Cell Project. Haematologica 2018;103:1191–7. 29599200 10.3324/haematol.2017.186577 PMC6029527

[4] Vose J Armitage J Weisenburger D. International T-Cell Lymphoma Project. International peripheral T-cell and natural killer/T-cell lymphoma study: pathology findings and clinical outcomes. J Clin Oncol 2008;26:4124–30. 18626005 10.1200/JCO.2008.16.4558

[5] Vose JM. Peripheral T-cell lymphoma: novel backbone. Blood 2018;131:375–6. 29371206 10.1182/blood-2017-11-817734

[6] Sandell RF Boddicker RL Feldman AL. Genetic landscape and classification of peripheral T Cell lymphomas. Curr Oncol Rep 2017;19:28. 28303495 10.1007/s11912-017-0582-9 PMC5517131

[7] Broccoli A Zinzani PL. Peripheral T-cell lymphoma, not otherwise specified. Blood 2017;129:1103–12. 28115372 10.1182/blood-2016-08-692566

[8] Sharma M Aronow WS O’Brien M. T cell lymphoma presenting as esophageal obstruction and bronchoesophageal fistula. Med Sci Monit 2011;17:CS66–69. 21629192 10.12659/MSM.881797 PMC3539550

[9] Schmitz N Trümper L Ziepert M. Treatment and prognosis of mature T-cell and NK-cell lymphoma: an analysis of patients with T-cell lymphoma treated in studies of the German high-grade non-hodgkin lymphoma study group. Blood 2010;116:3418–25. 20660290 10.1182/blood-2010-02-270785

[10] Zhang X-M Li Y-X Wang W-H. Survival advantage with the addition of radiation therapy to chemotherapy in early stage peripheral T-cell lymphoma, not otherwise specified. Int J Radiat Oncol Biol Phys 2013;85:1051–6. 23021436 10.1016/j.ijrobp.2012.08.015

